# Combining microfluidics and RNA-sequencing to assess the inducible defensome of a mushroom against nematodes

**DOI:** 10.1186/s12864-019-5607-3

**Published:** 2019-03-25

**Authors:** Annageldi Tayyrov, Claire E. Stanley, Sophie Azevedo, Markus Künzler

**Affiliations:** 1 Institute of Microbiology, Department of Biology, ETH Zürich, Vladimir-Prelog-Weg 4, CH-8093 Zürich, Switzerland; 2 Agroecology and Environment Research Division, Agroscope, Reckenholzstrasse 191, CH-8046 Zürich, Switzerland

**Keywords:** Transcriptomics, Fungal defense, RNA sequencing, Microfluidics, 16S rRNA sequencing, Pore-forming toxins

## Abstract

**Background:**

Fungi are an attractive source of nutrients for predators. As part of their defense, some fungi are able to induce the production of anti-predator protein toxins in response to predation. A previous study on the interaction of the model mushroom *Coprinopsis cinerea* by the fungivorous nematode *Aphelenchus avenae* on agar plates has shown that the this fungal defense response is most pronounced in the part of the mycelium that is in direct contact with the nematode. Hence, we hypothesized that, for a comprehensive characterization of this defense response, an experimental setup that maximizes the zone of direct interaction between the fungal mycelium and the nematode, was needed.

**Results:**

In this study, we conducted a transcriptome analysis of *C. cinerea* vegetative mycelium upon challenge with *A. avenae* using a tailor-made microfluidic device. The device was designed such that the interaction between the fungus and the nematode was confined to a specific area and that the mycelium could be retrieved from this area for analysis. We took samples from the confrontation area after different time periods and extracted and sequenced the poly(A)^+^ RNA thereof. The identification of 1229 differentially expressed genes (DEGs) shows that this setup profoundly improved sensitivity over co-cultivation on agar plates where only 37 DEGs had been identified. The product of one of the most highly upregulated genes shows structural homology to bacterial pore-forming toxins, and revealed strong toxicity to various bacterivorous nematodes. In addition, bacteria associated with the fungivorous nematode *A. avenae* were profiled with 16S rRNA deep sequencing. Similar to the bacterivorous and plant-feeding nematodes, Proteobacteria and Bacteroidetes were the most dominant phyla in *A. avenae*.

**Conclusions:**

The use of a novel experimental setup for the investigation of the defense response of a fungal mycelium to predation by fungivorous nematodes resulted in the identification of a comprehensive set of DEGs and the discovery of a novel type of fungal defense protein against nematodes. The bacteria found to be associated with the fungivorous nematode are a possible explanation for the induction of some antibacterial defense proteins upon nematode challenge.

**Electronic supplementary material:**

The online version of this article (10.1186/s12864-019-5607-3) contains supplementary material, which is available to authorized users.

## Background

In their ecological environment, fungi are exposed to a variety of organisms including predators that use fungi as a food source [1]. Significant predators of fungi include mollusks, insects, collembola and nematodes [1–3]. During evolution, fungi have developed several defense lines to protect themselves against these predators. These defense lines can be categorized into three groups; physical barriers [4, 5], secondary metabolites [6, 7] and peptides/proteins [8, 9]. Amongst defense proteins, biotin-binding proteins [10], protease inhibitors [11], pore-forming proteins [12], ribotoxins [13], and lectins [14] have been characterized. While defense proteins against competitors tend to be secreted, protein toxins against predators are usually stored in the cytoplasm [15].

Given the diversity of antagonists that fungi need to combat, the list of characterized fungal defense proteins is most likely not complete. Hence, determining the complete set of defense effectors (defensome) of a fungus would help to attain a clearer picture of the chemical defense capacity of these organisms. Earlier studies have shown that genes encoding for fungal defense effectors can be induced upon challenge of a fungus with its antagonists and, thus, it is possible to identify novel defense effectors on the basis of differential gene expression [16–19].

Previous studies on the antagonistic interaction between the coprophilous model basidiomycete *Coprinopsis cinerea* and the fungivorous nematode *Aphelenchus avenae* have shown that induction of fungal defense genes is limited to the part of the mycelium that is in direct contact with the worm [20]. Hence, for the identification of the complete set of genes of a multicellular fungus that is differentially expressed upon challenge with this type of predator, it is crucial to use an experimental setup that maximizes the area of direct interaction between the mycelium and the nematode and allows retrieval of the fungus from that area. In this respect, previously used co-cultivation methods on agar plates are not ideal [19]. As a solution, a tailor-made microfluidic device, referred to as fungus-nematode interaction (FNI) device, that allows confrontation between the fungus and the fungivorous nematode in a confined area and retrieval of the organisms from this area for analysis was developed. We used this setup to identify the inducible defensome of *C. cinerea* against the fungivorous nematode *A. avenae* by RNA-sequencing. The induced defensome contained several putative defense proteins, including a previously undetected putative β-pore-forming toxin which showed strong nematotoxicity when heterologously expressed in *Escherichia coli* and tested for toxicity towards several bacterivorous nematodes. This result suggests that the bacterial cytolysin-like toxin of *Coprinopsis cinerea* may represent a novel type of fungal effector proteins against nematodes. Furthermore, we identified the composition of bacteria associated with the nematode *Aphelenchus avenae*. To the best of our knowledge, this is the first study to profile the microbiome of a fungivorous nematode.

## Methods

### Strains and cultivation conditions

The self-compatible AmutBmut strain of *Coprinopsis cinerea* was maintained on YMG (0.4% (*w*/*v*) yeast extract, 1% (w/v) malt extract, 0.4% (w/v) glucose, 1.5% (w/v) agar) at 37 °C in humid dark chambers. The nematode *Aphelenchus avenae* was propagated on a sporulation-deficient strain (BC-3) [21] of the ascomycetous mold *Botrytis cinerea* on malt extract agar medium (MEA) supplemented with additional 15 g/L agar and 100 μg/ml chloramphenicol at 20 °C. All organisms used in this study are listed in Additional file 1.

### Challenge experiment setup and total RNA extraction

**Fig. 1:**
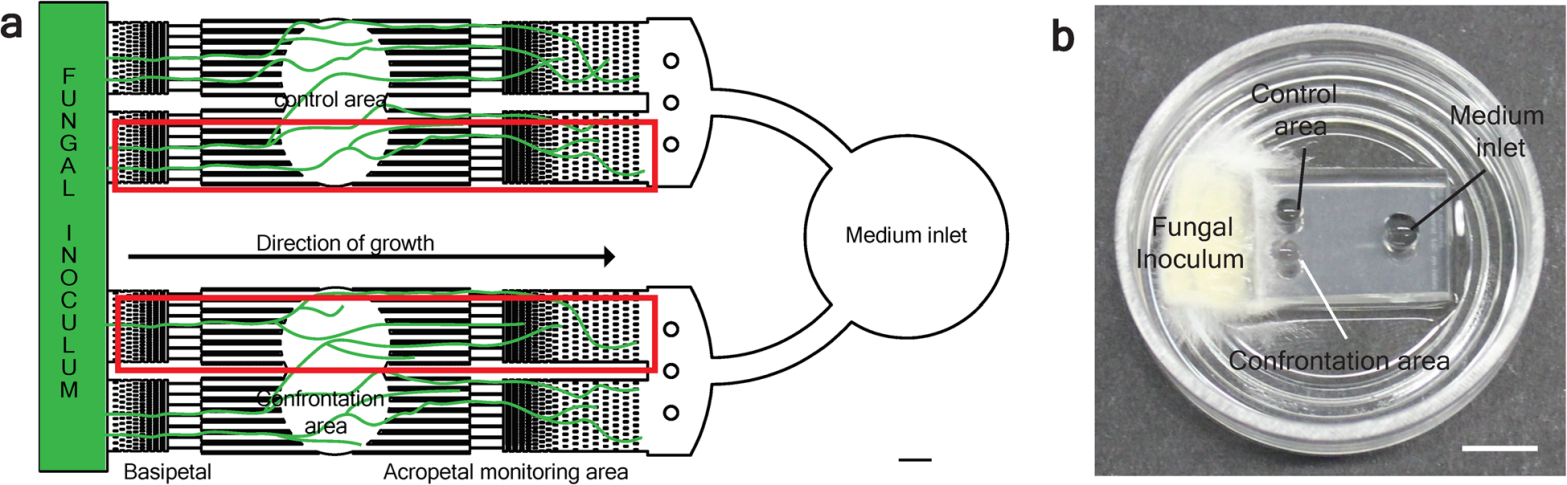
Fungal-nematode-interaction (FNI) microfluidic device*. **a** Scheme showing the overall design of the FNI device comprising confrontation area, control area and medium inlet. Scale bar, 500 μm. **b** Photograph of the FNI microfluidic device that was inoculated with *C. cinerea* and incubated for 18 h at 37 °C. Scale bar, 5 mm. *Reprinted from “Bidirectional Propagation of Signals and Nutrients in Fungal Networks via Specialized Hyphae”, Stefanie S. Schmieder, Claire E. Stanley, Andrzej Rzepiela, Dirk van Swaay, Jerica Sabotič, Simon F. Nørrelykke, Andrew J. deMello, Markus Aebi, Markus Künzler, Copyright (2019), with permission from Elsevier [20]

The fungus-nematode challenge was performed in tailor-made microfluidic device as previously described [22] with some modifications (Fig. 1a and b) [20]. Briefly, freshly prepared microfluidic devices were filled with 100 μl liquid YMG medium and inoculated with a small *C. cinerea* plug that was cut from precultivated mycelium on YMG agar plates at 37 °C for three days. The inoculated devices were incubated at 37 °C for 30 h in a humid dark chamber. The nematodes were harvested from *B. cinerea* plates by Baermann funneling [23] and purged by letting them crawl on water agar plates containing 50 μg/ml gentamycin, 50 μg/ml nystatin and 100 μg/ml ampicillin to eliminate potential fungal and bacterial contaminants. Around ten purged worms were added to the confrontation areas of the microfluidic devices at three different time points. Adding the nematodes at the different time points made it possible to harvest all samples at the same time having the fungus cultivated for the same but challenged for different time periods (Additional file 2). Cocultivations were performed in three biological replicates at 20 °C in the dark. Mycelium was retrieved from the confrontation area upon three different confrontation periods i.e. 2 h, 8 h and 20 h, in the presence and absence (i.e. controls) of the predator nematode, using a sterile pipette. RNA of all samples was extracted using the Norgen RNA extraction kit according to the manufacturer’s protocol (Norgen Biotek Corporation, Canada).

### RNA-sequencing and bioinformatic analysis

The concentration and quality of the isolated RNA samples were determined using a Qubit® (1.0) Fluorometer (Life Technologies, California, USA) and a Bioanalyzer 2100 (Agilent, Waldbronn, Germany), respectively. Samples with a 260 nm/280 nm ratio between 1.8–2.1 and a 28S/18S ratio within 1.5–2 were further processed. Transcriptome library preparation was performed at the Functional Genomics Center Zurich (FGCZ) using the TruSeq RNA Sample Prep Kit v2 (Illumina Inc., California, USA) following the manufacturers’ protocols. Briefly, total RNA samples (100–1000 ng) were enriched for poly(A)^+^ RNA and then reverse-transcribed into double-stranded cDNA. The cDNA sample was fragmented, end-repaired and polyadenylated before ligation of TruSeq adapters containing the index for multiplexing. Fragments containing TruSeq adapters on both ends were selectively enriched with PCR. The quality and quantity of the enriched libraries were validated using Qubit® (1.0) Fluorometer and the Caliper GX LabChip® GX (Caliper Life Sciences, Inc., USA). Sequencing was performed on the Illumina HiSeq 2000 single end 100 bp using the TruSeq SBS Kit v3-HS (Illumina, Inc., California, USA).

The data was analyzed using the SUSHI platform [24], which was developed at FGCZ and supports variety of open source ‘omics’ data analysis packages. In brief, reads were aligned with STAR aligner [25] with the additional options as described before [26]. The *C. cinerea* AmutBmut genome (2013-07-19) together with its latest annotation v1.0 (2016-09-12, filtered gene models) from the Joint Genome Institute (JGI) were used as a reference [27]. The Bioconductor featureCounts function were run to compute expression counts [28]. DESeq2 analysis package was used for differential expression analysis [29].

Genes were considered significantly differentially expressed if they had at least a four-fold change in nematode-treated samples compared to the control samples and a false discovery rate (FDR) of at most 0.05. In addition, 10 reads/locus were taken as a minimal threshold for gene expression. SignalP v4.1 [30], and TMHMM v.2.0 [31] were used for the detection of signal peptides and transmembrane domain of proteins, respectively. PFAM-A v31 [32] and Gene Ontology (GO) [33] databases were used for putative functional annotations. DEGs were classified into functional categories using Blast2GO with default parameters [34].

### Analysis of nematode-associated bacteria using 16S rRNA sequencing

*A. avenae* was cultured on MEA agar plates preseeded with *Botrytis cinerea* as food. After two weeks, nematodes were harvested by Baermann funneling [23] under sterile conditions. Nematodes were ground in liquid nitrogen using a pellet pestle in 1.5 ml sterile Eppendorf tubes. Thereafter, the total (nematode+bacteria) genomic DNA was isolated with E.Z.N.A. Mollusc DNA Kit (Norcross, OMEGA, USA) based on manufacturer’s instruction.

The V4 hypervariable region of the bacterial 16S rRNA gene was amplified using the primer pair 515F (5′- GTG CCA GCM GCC GCG GTA A -3′) and 806R (5′- GGA CTA CHV GGG TWT CTA AT -3′). The prepared PCR libraries were sequenced on the Illumina MiSeq platform using v2 nano 500 cycles kit. The generated paired-end reads were first filtered using Illumina’s chastity filter and then de-multiplexed and trimmed using Illumina’s real time analysis software. The reads were subjected to quality control by FastQC version 0.11.5. The locus-specific primers were removed using cutadapt v1.14. Reads with untrimmed adaptors were discarded. Merging of the forward and reverse reads, considering a minimum overlap of 15 bases, were done using the software USEARCH version 10.0.240 [35]. A maximum of one expected error per merged read was allowed and reads with ambiguous bases were discarded. The merged reads were denoised using the UNOISE algorithm of USEARCH. Next, operational taxonomic units (OTUs) were formed, and singletons and chimeras were discarded in the process. The formed OTUs were searched using the reference sequences of the RDP 16S database [36]. Taxonomy prediction was performed using the SINTAX algorithm (min_threshold = 0.7) of USEARCH. Alpha diversity calculations and rarefaction analysis were performed with the software phyloseq v1.16.2 [37]. The illumina sequencing and the subsequent data analysis described in this section were performed by Microsynth AG (Balgach, Switzerland). Generated nucleotide sequences were deposited at NCBI SRA database under the accession number SRP152111.

### Cloning and heterologous expression of P450139-encoding cDNA

First strand cDNA was synthesized from 1 μg total RNA of nematode-induced *C. cinerea* AmutBmut using Transcriptor first strand cDNA synthesis kit (Roche) following the manufacturer’s protocol. The coding region of P450139 (JGI MycoCosm ProteinID) was amplified from first strand cDNA using the P450139forNdeI and P450139revNotI primer pair (Additional file 3). The PCR product was cloned into expression vector pET24b (Novagen) using *Not*I and *Nde*I restriction sites. The constructed plasmid was transformed into the chemo-competent *E. coli* DH5α cells. The sequence of the plasmid was verified by DNA-sequencing and the plasmid was transformed into *E. coli* BL21 cells for expression. Colonies were cultured in LB medium containing 50 mg/l kanamycin at 37 °C until OD600 0.5 and induced with 0.5 mM isopropyl β-D-1-thiogalactopyranoside (IPTG) at 16 °C for 24 h. Expression and solubility of the protein were assessed as previously described [38].

### Toxicity of P450139 against nematodes and insects

To assess the toxicity of P450139 protein against nematodes, eggs were isolated from five species of bacterivorous nematodes (*Caenorhabditis elegans, Caenorhabditis briggsae, Caenorhabditis tropicalis, Halicephalobus gingivalis, Pristionchus pacificus*) (Additional file 1) based on wormbook guidelines [39]. The nematodes were precultivated on NGM (Nematode growth medium) plates (2.5 g/L peptone, 50 mM NaCl, 1.7% agar and 13 mM cholesterol) seeded with *E. coli* OP50. Eggs were isolated by bleaching gravid hermaphrodites and hatched on 1% water agar plates overnight at 20 °C. Synchronized L1 stage larvae were collected in PBS and adjusted to 10–15 nematodes/10 μl. *E. coli* BL21 containing plasmids directing the heterologous expression of P450139, CGL2 (*Coprinopsis cinerea* galectin 2) and “empty” vector (EV) were preinduced with IPTG as described above. The CGL2-expressing plasmid and EV were used as positive and negative controls, respectively. 20 μl of number-adjusted L1 nematodes were incubated in 100 μl of PBS adjusted to OD_600_ 2.0 of preinduced *E. coli* BL21 cells in a 96 well plate as described before [38]. The plate was incubated for 48 h at 20 °C for all nematodes except for *H. gingivalis* (72 h). The percentage of nematodes that developed into L4 larvae or adulthood were counted. All treatments were performed in tree biological replicates. Dunnett’s multiple comparisons test was used for statistical analysis. Furthermore, toxicity of P450139 towards *Aedes aegypti* larvae was tested as previously described [38].

### Tagging and purification of P450139

To perform the in vitro hemolytic activity assay, P450139 was tagged at N-terminus with 8 His-residues and purified on Ni-NTA columns as described previously [14]. In brief, the parental P450139-encoding pET-24b plasmid was amplified with P450139forHis8 and P450139revHis8 primers (Additional file 3). The PCR product was treated with DpnI to eliminate the methylated template plasmid. 5 μl of DnpI-treated PCR product was transformed into *E. coli* DH5a for plasmid purification and subsequent sequencing. The plasmid was transformed into *E. coli* BL21 for protein expression and purification. The protein was expressed as described above for the untagged variant of the protein. The bacterial cells were harvested by centrifugation and resuspended in cold lysis buffer (PBS, 20 mM imidazole, pH 8.5) before being lysed using French press. The cell lysate was spun at 16000 rpm for 30 min at 4 °C and the supernatant containing the soluble fraction was incubated with Ni-NTA beads (Macherey-Nagel) for 1 h at 4 °C. The protein bound to the beads was washed with lysis buffer and subsequently eluted with lysis buffer supplemented with 250 mM imidazole. The eluate was desalted and concentrated on PD10 column (GE healthcare).

### Hemolysis assay for P450139

Defibrinated horse blood (Thermo Fisher) was washed three times with PBS by spinning down for 15 min at 4500 g in between. Two different concentrations (2 and 0.2 mg/mL) of the purified P450139 protein was added to 100 μl of the washed blood cells. The hemolysis reaction was incubated for one hour at room temperature followed by centrifugation for 10 min at 2400 g. The supernatant was transferred to a clear bottom 96-well plate and absorbance was measured at room temperature using a microplate reader (SpectraMax Plus, Molecular Devices Corp.) at OD_450_. Bovine serum albumin (Sigma-Aldrich®) and 10% triton™ X-100 (Sigma-Aldrich®) were used as negative and positive controls, respectively. All treatments were performed in triplicates.

## Results

### Differentially expressed genes (DEGs) of *C. cinerea* in response to cocultivation with *A. avenae* for different time periods

To determine the defensome of a fungus against a fungivorous nematode, we sequenced the transcriptome of *C. cinerea* challenged with *A. avenae* using the FNI device (Fig. 1a and b)*.* In order to gain an insight into the dynamics of the fungal defense response, DEGs were analyzed after three different time periods of cocultivation of the fungal mycelium with the fungivorous nematode. Strand specific RNA-seq libraries were prepared for three biological replicates of each cocultivation period and sequenced using the Illumina HiSeq 2000 platform.

**Fig. 2:**
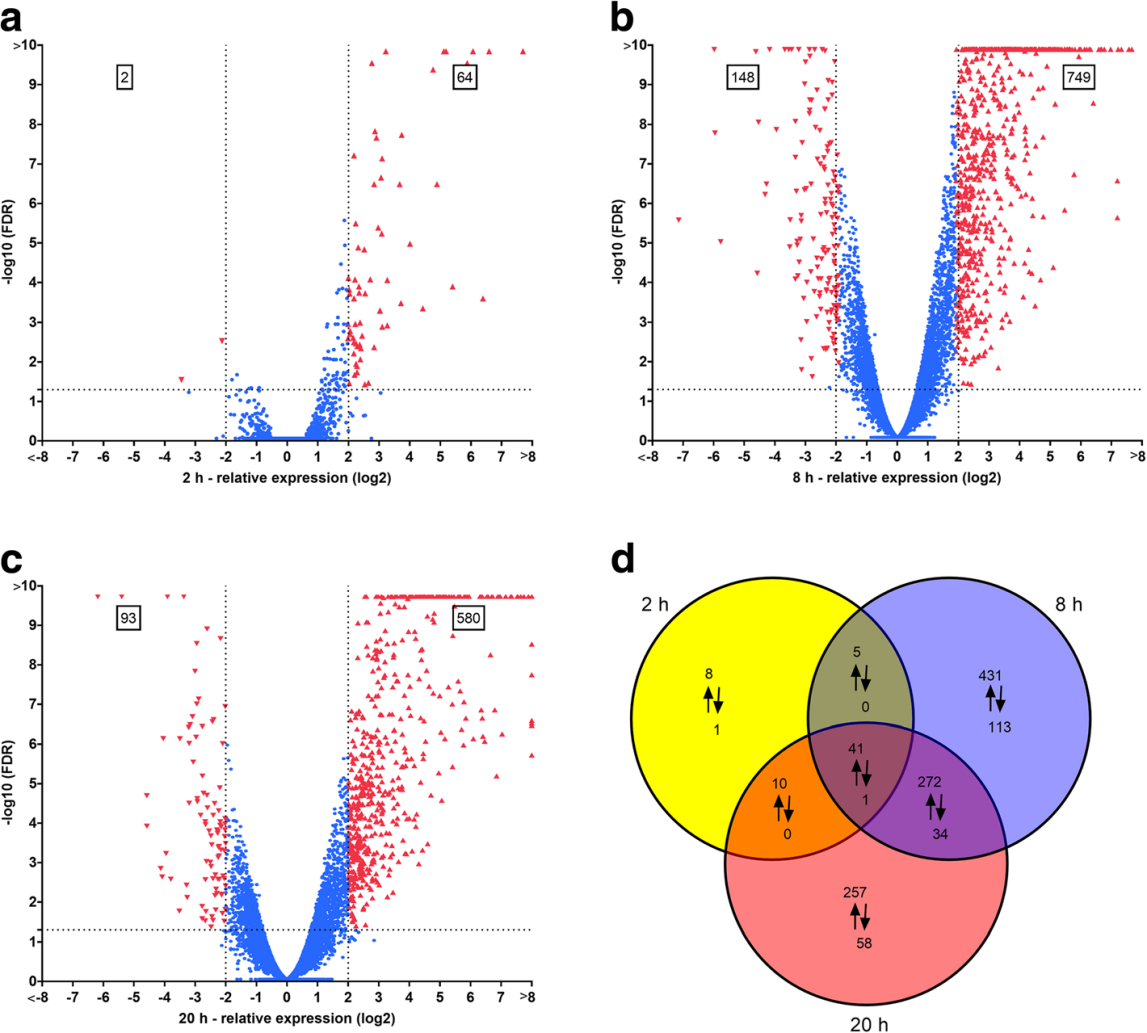
Differentially expressed genes (DEGs) of *C .cinerea* challenged by *A. avenae* for different time periods. Volcano plots showing DEGs in relation to FC (Fold change) and FDR (False discovery rate) for **a** 2 h nematode challenge vs. unchallenged control, **b** 8 h nematode challenge vs. unchallenged control, **c** 20 h nematode challenge vs. unchallenged control. Each gene is represented with a single data point. Genes with at least four fold change and FDR < 0.05 are colored red and considered significantly upregulated (upward triangles) or downregulated (downward triangles). **d** Venn diagram showing significantly upregulated (↑) and downregulated (↓) genes due to nematode challenge for each time point vs. unchallenged control. Overlapping areas represent DEGs common to different time points

For every sample, over 30 million reads (100 bp) were generated, and 83–88% of all generated reads mapped to the 37.5 Mb genome of *C. cinerea* AmutBmut. The percentage of the genomic features with at least 10 mapped reads were around 80% for all samples (Additional file 4). The *C. cinerea* genome contains 14,242 predicted protein-encoding genes according to the JGI MycoCosm (May 2018) [40]. Thus, the 80% roughly represent 11,400 of the predicted protein-encoding genes. These genes were considered transcribed. The significance of differential gene expression of *C. cinerea* was assessed by comparing the DESeq2 normalized read values of the various samples. Upon nematode challenge of 2 h, 8 h and 20 h, 66, 897 and 673 genes were significantly differentially expressed (FDR ≤ 0.05 AND |Fold change| ≥ 4), respectively, when compared to the control. Most of the DEGs (1024 out of 1229) were upregulated while only 17% of the DEGs were downregulated in the presence of nematodes (Fig. 2a-d). Roughly, 50% of DEGs (549) could be assigned to a PFAM domain (Additional file 5). The majority of the annotated genes were assigned as a DUF (domain of unknown function). Visualization of GO terms shows the the process ‘response to toxic substance’ is enriched in the upregulated set of the genes while oxidation-reduction processes were highly enriched in both the upregulated and downregulated set of genes (Additional file 6). In agreement with their biological processes, genes coding for proteins with oxidoreductase activity were considerably enriched among the molecular functions for both sets of differentially expressed genes (Additional file 6). In addition, the upregulated set of the genes included previously characterized cytoplasmic nematotoxic proteins CGL2, CCL2 (*Coprinopsis cinerea* lectin 2) and CCTX2 (*Coprinopsis cinerea* toxin 2) (Additional file 5) [14, 19]. The transcriptomics data is available at the ArrayExpress database under the accession number E-MTAB-7005.

### Comparison between microfluidics- and agar-based challenges of *C. cinerea* with *A. avenae*

**Fig. 3:**
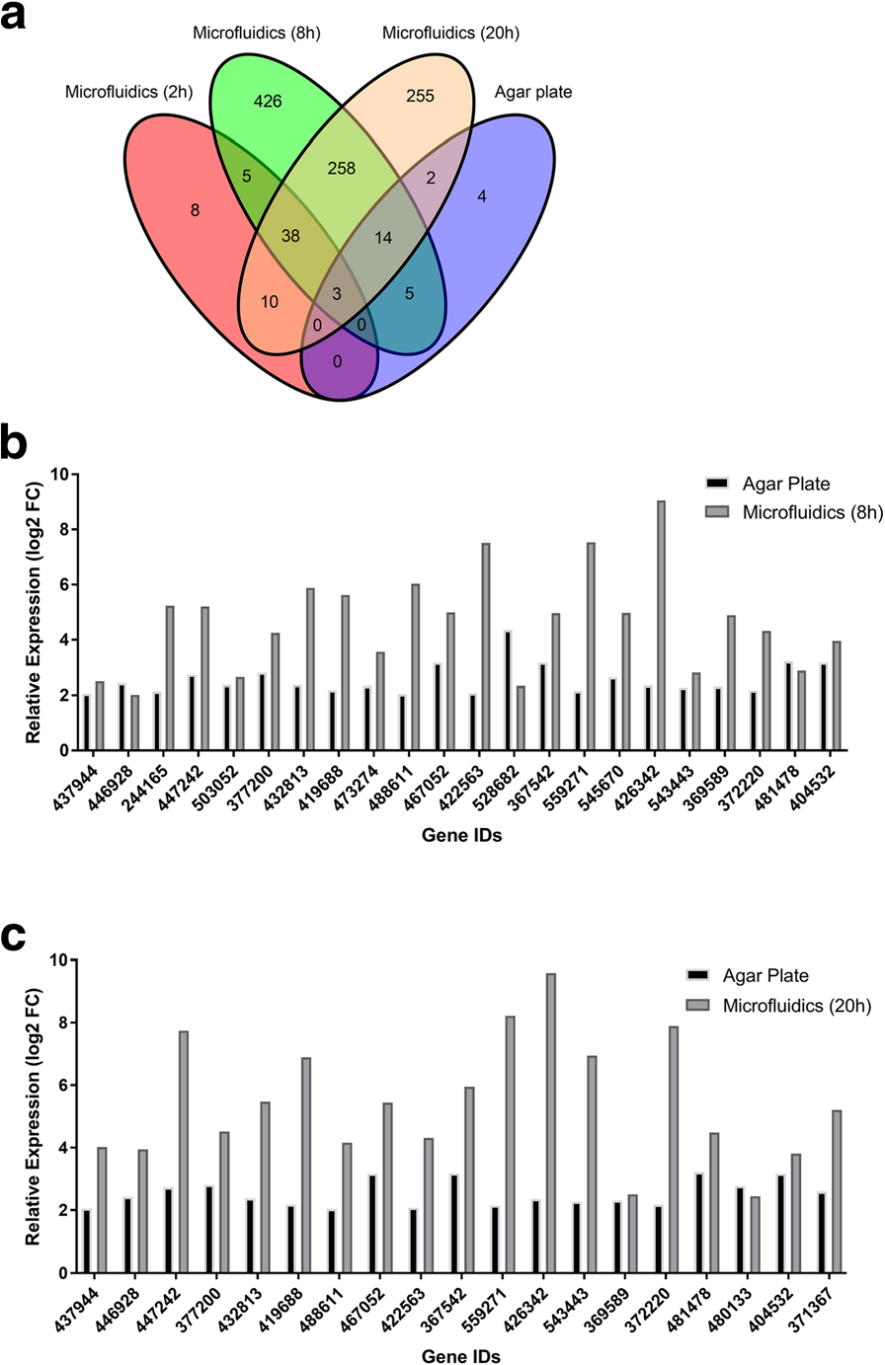
Comparison between microfluidics and agar-based confrontation of *C. cinerea* and *A. avenae*. **a** Venn diagram representing significantly upregulated genes for two different setups (microfluidic device and agar plate). **b** 20 out of 22 DEGs shared between microfluidics-8 h and agar plate assay have higher relative expression in microfluidics compared to agar plate. **c** 18 of 19 DEGs shared between microfluidics-20 h and agar plate assay reveal a higher relative expression in the microfluidic devic . Genes with fold change of ≥4 and FDR ≤ 0.05 considered as significantly induced

We hypothesized, based on a previous microfluidics study [20], that performing the fungus-nematode challenge in the FNI device would allow us to collect and analyze samples from sections of the fungal mycelium that are highly induced. In agreement with this hypothesis, the transcriptome analysis of the microfluidics-based challenge yielded, in comparison to a previous agar-based challenge [19], a substantially higher number of differentially expressed genes (Fig. 3a). Both studies included 3 replicates and were of comparable sequence depth (current microfluidics-based study: 33.5 million mapped reads/sample, previous agar plate-based study [19]: 41.8 million mapped reads/sample). Due to the slower response rate of the fungus on agar plate, the exposure time was longer (72 h) in the previous assay. 24 out of 28 of the upregulated genes in the agar plate-based assay were also found to be upregulated in microfluidics-based assay. In agreement with above hypothesis, the fold change of almost all genes that had already been found to be differentially expressed on agar, was higher when the confrontation was performed in the microfluidic device (Fig. 3b and c).

### Diversity of *Aphelenchus avenae*-associated bacteria

**Fig. 4:**
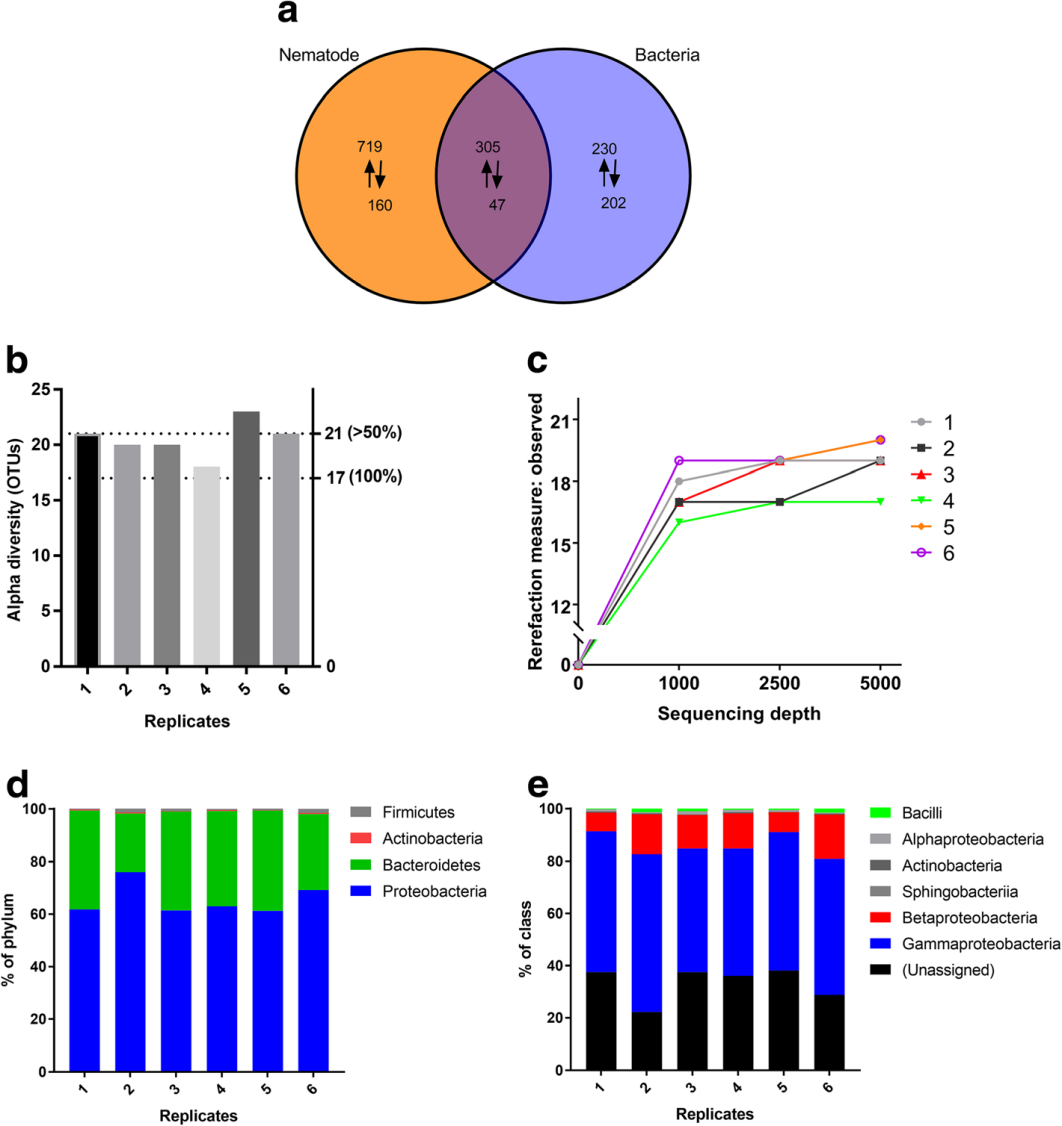
Associated bacteria of fungivorous nematode *A. avenae*. **a** Venn diagram showing significantly upregulated (↑) and downregulated (↓) genes of *C. cinerea* in response to nematode (present study) and bacteria (previous study) challenges. Genes with fold change of ≥4 and FDR ≤ 0.05 considered as significantly upregulated while genes with fold change of ≤ − 4 and FDR ≤ 0.05 considered as significantly downregulated **b** Assigned OTU numbers for associated bacteria of *Aphelenchus avenae* for each biological replicate. **c** Rarefaction analysis of observed OTUs of nematode-associated bacteria. The relative abundance of the bacterial composition of the nematode at phylum (**d**) and class (**c**) levels

The obtained 1229 DEGs of the current study were compared to our previous RNA-sequencing data (784 DEGs) of *C. cinerea* mycelium challenged with *E. coli* and *B. subtilis* [26]. More than half of the genes that were upregulated due to bacteria-challenge were also found to be upregulated in our current nematode-challenge dataset (Fig. 4a). Based on these findings, we analyzed the fungivorous nematode for naturally associated bacteria which could be responsible for the upregulation of antibacterial genes in *C. cinerea*. While there are several studies on associated bacteria of bacterivorous [41, 42], animal parasitic [43] and plant pathogenic nematodes [44–52], to our knowledge there is only one study on the diversity of bacteria associated with a fungivorous nematode *Aphelenchus* sp. [53]. However, in the latter study, only five OTUs could be detected.

In this study, we determined the diversity of the bacteria associated with *A. avenae* by high-throughput sequencing of their 16S rDNA. The nematodes were cultivated on *Botrytis cinerea* for two weeks. Thereafter, the worms were isolated under sterile conditions and total DNA was extracted. The libraries were prepared from six biological replicates. Sequencing was performed on the Illumina MiSeq platform. The analyses yielded a total of 47,672 high quality reads with an average of 7945 reads per sample. The reads were clustered into OTUs in USEARCH. The distributed numbers of identified OTUs ranged from 18 to 23 for six samples. 17 of the distributed OTUs were common for all samples while 21 OTUs were found in more than half of the samples (Fig. 4b). The rarefaction curves for all samples were flattened within the obtained sequencing depth (Fig. 4c), indicating that the sampling was reasonable and able to represent the majority of the bacterial community associated with the fungivorous nematode.

The generated OTUs were searched against reference sequences of the RDP 16S database, and predicted taxonomical units were assigned (Additional file 7). The biological replicates were generally consistent (Additional file 8). Bacteria associated with the fungivorous nematode were classified into four taxonomical phyla where the predominant phyla of all samples were Proteobacteria and Bacteroidetes (Fig. 4d). The Proteobacteria were mostly Gammaproteobacteria and Betaproteobacteria (Fig. 4e). At genus level, *Coxiella*, *Halomonas*, *Escherichia/Shigella*, *Herbaspirullum* altogether make up the majority of the assigned bacteria whereas the proportion of unassigned OTUs was 34% on average per sample at this taxonomic level (Additional file 8).

### P450139 is a nematode-inducible nematotoxic protein with a putative hemolysin domain

**Fig. 5:**
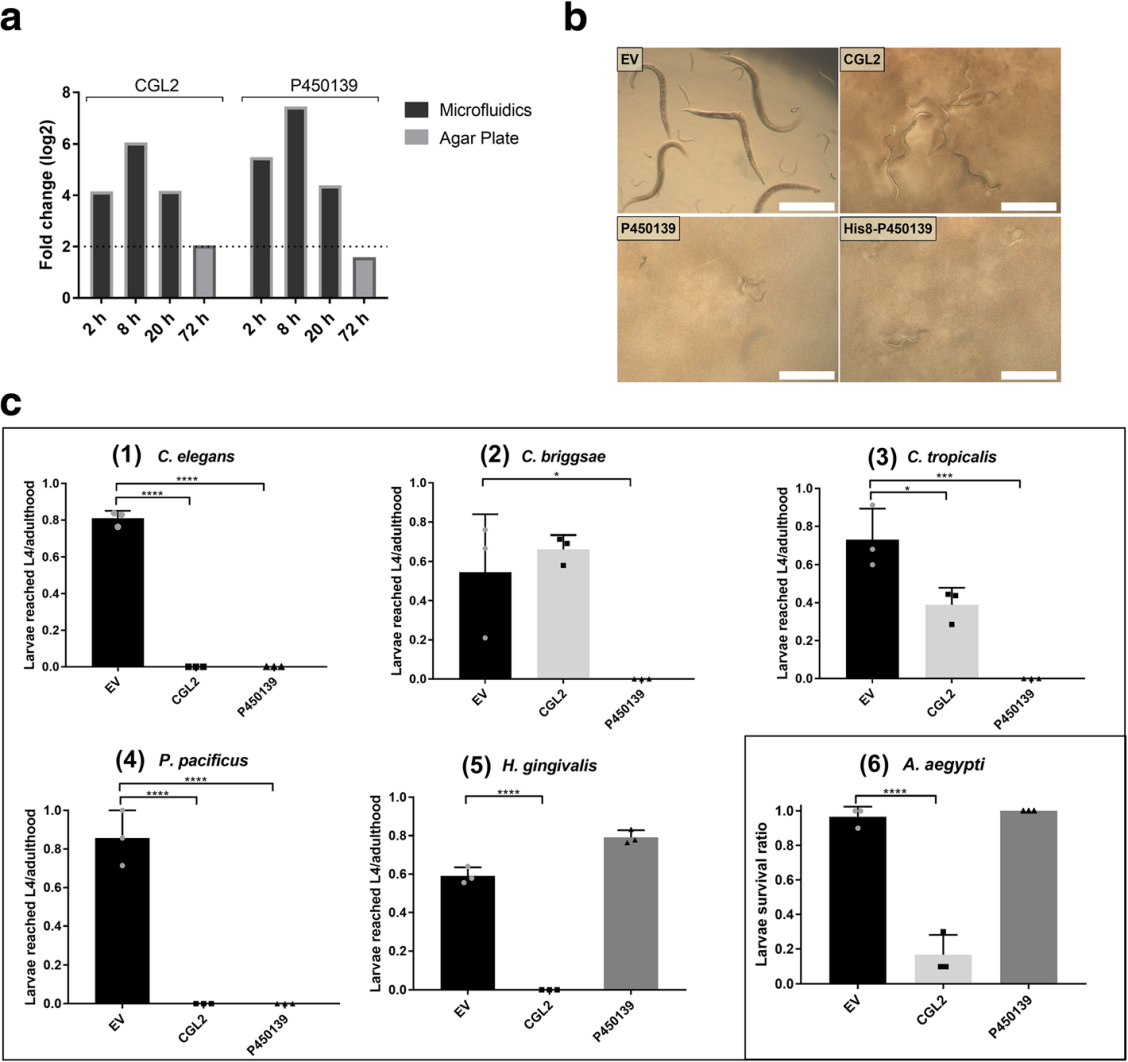
P450139 is an inducible nematotoxic protein with a putative hemolysin domain. **a** The *C. cinerea* gene coding for P450139 is significantly induced upon nematode challenge in the microfluidic setup in a similar manner to the previously identified nematotoxic lectin, CGL2. **b** Phase-contrast micrographs of *C. elegans* fed with IPTG-induced *E. coli* BL21 for 72 h, containing either an ‘empty’ vector (EV) or expressing CGL2, P450139 or P450139_8His. Scale bar = 500 μm. **c** The toxicity of the P450139 protein was assessed against five different species of bacterivorous nematodes **(1–5)** and larvae of *Aedes aegypti* **(6)***.* IPTG-induced *E.coli Bl21* bearing either the previously characterized nematotoxic protein CGL2 or an empty vector (EV) were used as positive and negative controls, respectively. Dunnett’s multiple comparisons test was used for statistical analysis. Error bars represent standard deviation of three biological replicates**.** ns**:** not significant, *p < 0.05, **p < 0.01, ***p < 0.001, ****p < 0.0001 vs. EV

The *C. cinerea* gene coding for the predicted protein P450139 showed a similar expression pattern as the previously characterized nematotoxic lectin CGL2 (Fig. 5a), suggesting that the gene products may have similar functions. Furthermore, a homology search at HHpred (Homology detection & structure prediction by HMM-HMM comparison) [54] indicated a similarity of the protein to bacterial β-pore-forming toxins, i.e. leukocidins (*Staphylococcus aureus*) [55] and hemolysin (*Vibrio cholera*) [56], as top hits with significantly high probabilities (Table 1). Based on this data, the cDNA of P450139 from *C. cinerea* was cloned and heterologously expressed in *E. coli* for further investigation. For the purification of the protein, an 8His- tag was introduced at the N-terminus of P450139. The heterologous expression and solubility of the untagged and tagged proteins were evaluated. Both protein variants were well expressed but their solubility was low (Additional file 9).

**Table 1:** The P450139 amino acid sequence was analyzed using HHpred server. Similarities were found for internal 75 amino acids (110–185)

#	Hit IDs	Annotation	Toxin type	Organism	Probability^a^	Template HMM
1	4IYA_A	S component leucocidin	Pore-forming toxin	*Staphylococcus aureus*	85.9	85–151 (292)
2	4Q7G_A	Leucotoxin LukDv	Pore-forming toxin	*Staphylococcus aureus*	71.8	104–173 (324)
3	4TW1_J	LUKG, LUKH; leukocidin;	Pore-forming toxin	*Staphylococcus aureus*	71.6	110–177 (324)
4	1XEZ_A	Hemolysin	Pore-forming toxin	*Vibrio cholerae*	70.5	258–332 (721)
5	7AHL_E	Alpha-hemolysin cytolytic protein	Pore-forming toxin	*Staphylococcus aureus*	66.1	83–154 (293)
6	4I0N_A	Necrotic enteritis toxin B	Pore-forming toxin	*Clostridium perfringens*	62.9	84–155 (289)

^a^Cut-off: > 50% probability

The soluble recombinant 8His-tagged P450139 protein was purified over Ni NTA. The purity of the purified protein was verified by SDS-PAGE and Coomassie blue staining (Additional file 9).

To check whether P450139 is toxic against nematodes, recombinant bacteria producing the protein were fed to five different species of bacterivorous nematodes. Impairment of nematode larval development was assessed after 48 h. *C. elegans, C. briggsae, C. tropicalis* and *P. pacificus* showed susceptibility to P450139 while the development of *H. gingivalis* larvae was not inhibited by the recombinant fungal protein (Fig. 5b and c).

To test whether P450139 is active against insects, its toxicity was assessed by feeding the recombinant bacteria to *A. aegypti* larvae. The mosquito larvae were not impaired in their development by this food, indicating that the fungal protein does not have insecticidal activity (Fig. 5c).

Finally, the hemolytic activity of P450139 towards horse erythrocytes was assessed. For this purpose, the cells were incubated with different concentrations of the 8His-tagged purified protein, pelleted and the OD_450_ of the supernatant was measured. P450139 did not cause any apparent lysis of horse erythrocytes (Additional file 9).

## Discussion

Here, we profiled the transcriptional response of the model fungus *C. cinerea* to the fungivorous nematode *A. avenae* using a microfluidics-based, tailor-made fungus-nematode interaction (FNI) device for the confrontation. In contrast to the previous challenge experiments [19] that were performed on agar plates, the microfluidic approach yielded a higher number of DEGs. In addition to the higher number of DEGs, the degree of differential expression of the DEGs that were found in both studies, was significantly higher in the microfluidic approach. This result is in accordance with the high expression level of the *cgl2p-dTomato* reporter gene in the fungal-nematode confrontation area of the fungal-nematode interaction (FNI) microfluidic device [20]. Previously characterized cytoplasmic nematotoxic proteins (CGL1, CGL2, CCTX2) [14, 19] were also found to be significantly upregulated in the microfluidic setup, confirming the presence of an antagonist-inducible defense response of fungi against their antagonists [17] and its reproducibility in the FNI microfluidic device. Taken together, these results show that the microfluidic assay is highly sensitive and provides significant advantages over the agar-based assay for assessing nematode-inducible defense responses of fungi. It should be noted, however, that the previous fungal-nematode challenge studies were performed only at a single time-point [19]. Thus, the number and the level of induced defense genes might not have reached their maximum expression level in these previous studies.

A comparison between the *C. cinerea* DEGs identified during this study and the DEGs of a previous study where *C. cinerea* was confronted with bacteria under submerse conditions [26] revealed considerable overlap between the two datasets. The DEGs that were identified in both studies, include some genes coding for characterized antibacterial defense proteins such as phage-type lysozymes and copsin paralogs. Various precautions were taken to avoid bacterial contaminations in the experiments e.g. occasional bleaching of gravid nematodes and starting nematode cultures from fresh eggs, cultivation of the nematodes on fungal plates containing antibiotics and elimination of potential contaminants after Baermann funneling by allowing nematodes to crawl on agar plates containing an antibiotic cocktail. In fact, several antibacterial proteins were found to be upregulated in previously performed fungal-nematode confrontation studies on agar plates [19]. These findings suggested the possibility of the involvement of nematode-associated bacteria in fungal-nematode interactions. The importance of the bacterial community of nematodes for the fitness and virulence of parasites was previously suggested in several studies [44, 45, 47, 48, 51]. Although there are several studies that have analysed the bacteria associated with bacterivorous [41, 42], plant pathogenic [44, 49, 50, 52], and animal parasitic [43] nematodes, currently no data concerning the bacterial communities associated with fungivorous nematodes is available. Taken together, these findings directed us to look into the bacterial diversity of our model fungivorous nematode, *Aphelenchus avenae,* using 16S rDNA amplification and sequencing*.*

The analysis of the six 16S rDNA libraries showed good consistency among replicates. The observed small variations among the biological replicates are most likely due to the sampling process as it was previously suggested that even the technical replicates can show significant differences due to the inherently introduced errors in amplicon sequencing-based detection [57]. Results showed that Proteobacteria and Bacteroidetes were the most dominant phyla. Previous studies on bacterial- [41, 53, 58] and plant-feeding [50, 53] nematodes showed very similar results, suggesting that – at least at higher taxonomic levels – bacteria associated with nematodes within different feeding groups are conserved. However, at lower taxonomic levels, such as the genus level, the diversity of bacteria starts to change significantly. Many of the most commonly found groups of bacteria, such as *Achromobacter* [49, 50], *Burkholderia* [52], *Herbaspirilium* [58], *Stenotrophomonas* [59], *Escherichia* [60], *Serratia* [45] and *Halomonas* [50], were shown in previous studies to be present in bacteria- and/or plant-feeding nematodes. Additionally, in this study, one of the most predominant genera was *Coxiella*, which has not previously been shown to form part of a nematode’s bacterial diversity. However, previous studies concerning bacteria associated with Arthropods showed that *Coxiella* are comprising 98% of the 16S rRNA sequences in both eggs and larvae of the cattle tick *Rhipicephalus microplus*, and the authors suggested that the bacteria provide a nutritional complement to the tick host [61]. In the same study, treatment of the tick with an antibiotic to kill its mutualistic symbionts resulted in developmental retardation of the tick. Very similar outcomes were observed for plant pathogenic nematodes when they were treated with antibiotics to eliminate their associated bacteria, albeit in this case different bacteria were involved [45]. Although the exact roles of these bacteria in nematodes remain largely unknown, they are, as suggested by previous studies [42, 51], likely to be important with regard to nematode fitness and might be involved in protecting the host against potential pathogens and enzymatic digestion of ingested food.

It is important to note that due to the surface sterilization treatments of the nematodes, we suspect that mainly the internal bacteria of the nematodes were detected. However, we cannot exclude that some bacteria might have originated from the surface of the nematodes. It has been previously shown that nematodes can carry bacteria in their eggs [43] and on their cuticle [62]. Since the entire nematode is used for DNA-extraction, we cannot make a statement in this regard. In future studies, it would be exciting to investigate the spatial distribution of the identified bacteria in the nematode.

Identification of a fungal defense proteins by challenge of the fungus with predatory nematodes and analysis of differential gene expression was one of the main goals of this study. As part of this process, we examined the genes of *C. cinerea* that were highly induced in an effort to identify novel nematotoxic defense proteins. For this purpose, we focused on the 8 h rather than the 20 h timepoint in order to avoid genes that were upregulated due to secondary effects of the nematode challenge. The DEGs comprised by the GO biological processes ‘response to toxic substance’ and ‘oxidation-reduction process’ may nevertheless fall into latter category as production of reactive oxygen species (ROS) has been identified as a conserved damage response of plants, animals and fungi to predator-induced cellular damage [63]. One of the highly induced genes, coding for P450139, however, showed, in addition to its strong upregulation at the 8 h timepoint, overall expression dynamics that were similar to previously characterized inducible nematotoxic proteins. Furthermore, structural homology searches suggested significant similarity to β-pore-forming bacterial toxins, namely staphylococcal leukocidins [55] and *Vibrio cholera* cytolysin (VCC) [56]. Interestingly, VCC had been shown to be toxic against nematodes [64], supporting our hypothesis that P450139 was a nematotoxic protein. Heterologously expression of P450139 in *E. coli* and testing the toxicity of these bacteria towards nematodes and insects revealed a strong toxicity of the protein against four of the five tested nematode species (*C. elegans*, *C. briggsae*, *C. tropicalis* and *P. pacificus*) whereas *H. gingivalis* was resistant. Interestingly, the four P450139-susceptible nematode species are phylogenetically more closely related to each other than to *H. gingivalis* [65]. This observation suggests the existence of a conserved target for P450139 in the closely related species, while this target is apparently lacking or not accessible in the resistant nematode species. Interestingly, the susceptibility pattern differed from the one of the CGL2 lectin, suggesting that the distribution of the targets of a protein toxin does not always correlate with the phylogeny of the target organisms. Above conclusions assume a direct effect of P450139 on the target organisms. Alternatively, the effect could be indirect in that the fungal protein releases endogenous toxins from *E. coli*. This issue and the toxicity against nematodes with a different feeding style such as plant pathogenic and fungivorous nematodes [66] will be subject of future investigations.Other bacterial pore-forming proteins that are structurally similar to P450139 lyse different blood cell types. *Vibrio cholera* cytolysin and staphylococcal leukocidin (LeukS-PV) lyse erythrocytes [67] and human leukocytes [68], respectively. The observed lack of lysis of the tested erythrocytes by P450139 under the applied assay conditions may be due to the lack of cofactors or activators. In fact, the bacterial homologue VCC undergoes activation with proteolytic cleavage prior to being able to assemble the pore forming structure [69]. Therefore, the lack of hemolytic activity could be due to the absence of the specific receptors, missing co-factors or a lack of prior activation. Nevertheless, the observed nematotoxic effects strongly support that P450139 is an inducible protein involved in the chemical defense of *C. cinerea* against nematodes. Interestingly, we couldn’t find any sequence homologous to P450139 within the NCBI non-redundant database or the JGI MycoCosm, suggesting that this protein toxin is so far unique to *C. cinerea*.

## Conclusion

In this study, we analyzed the genome-wide transcriptional response of a fungus upon challenge with a fungivorous nematode using a tailor-made microfluidic setup. Our results indicate that the current setup significantly improves the sensitivity of the analysis in comparison to standard agar-based confrontations.

The presence of a considerable diversity of bacteria associated with fungivorous nematodes and the induction of antibacterial defense genes in the fungus upon nematode predation suggest that nematode predation of a fungus is a tripartite rather than a bipartite interaction. Similar to plant defense against plant pathogenic nematodes, the induction of antibacterial defense genes might allow the fungus to defend itself against nematode predators in part by interfering with the microbiome of the nematode.

One of the identified nematode-induced *C. cinerea* genes, coding for P450139*,* was previously missed most likely due to the limited sensitivity of agar plate-based assays. This protein, having a predicted structural homology comparable to that of bacterial pore forming toxins, showed strong toxicity against four of the five bacterivorous nematodes tested, suggesting that P450139 is a part of the inducible armory of *C. cinerea* against predatory nematodes*.* The different specificities of the nematotoxins P450139 and CGL2 with regard to nematode species are probably a consequence of the different modes of action of these protein toxins and implies that the various nematotoxins of a specific fungus have complementary specificities, ensuring an efficient defense against a large number of different predators. We propose P450139 as a novel type of pore-forming protein toxins of *C. cinerea* based on its structural similarity to the well-studied *Staphylococcus aureus* and *Vibrio cholera* pore-forming toxins. The self-protection mechanism of the fungus towards this cytoplasmic protein toxin and its mode of action in nematodes remain to be elucidated.

## Additional files

Additional file 1: **Table S1.** Used organisms. Used organism in this study including strain and source information. (DOCX 13 kb)

Additional file 2: **Figure S1.** Nematode-fungus challenge experiment setup and RNA extraction. Microfluidic devices were inoculated with a small *C. cinerea* plug and incubated for 30 h. Around ten worms were added to the confrontation area of the microfluidic device at three different time points i.e. 2 h, 8 h and 20 h. The time point when nematodes were added are indicated with a red triangle. The control samples were incubated in the absence of the predator nematodes throughout. All samples were harvested at the same time point (t_0_) and used for total RNA extraction. Each treatment was performed in three biological replicates. (PNG 70 kb)

Additional file 3: **Table S2.** Primer list. List of the primers used in this study. (DOCX 12 kb)

Additional file 4 **Table S3.** RNA sequencing read counts and alignment statistics. As stated in the title. (DOCX 15 kb)

Additional file 5 RNA-seq analysis of *C. cinerea* mycelium challenged by *A. avenae*. The file includes all DEGs of the *C. cinerea* together with their relative and absolute expression values and PFAM and GO-term annotations (XLSX 5200 kb)

Additional file 6 **Figure S2.** Annotation of DEGs with Blast2GO into functional categories. All (a), upregulated (b) and downregulated (c) genes of *C. cinerea* due to nematode challenge were annotated with Blast2GO. Categories are grouped by biological process (BP) and molecular function (MF). Numbers indicate the number of genes assigned to each GO term. (PNG 1140 kb)

Additional file 7: OTU read numbers and taxonomic assignments. The file contains of discovered OTUs in each replicate, their percentage and numbers, and every assigned taxonomic units up until genus level. (XLSX 13 kb)

Additional file 8: **Figure S3.** Analysis of 16 rRNA-based associated bacteria of *A. avenae*. Description: (a) Heat map showing 16S rDNA based profile of *A. avenae* associated bacteria for each of the six biological replicates. The figure legend represents the percentage of assigned OTUs to each genus of bacteria. (b) The pie chart represents the composition of nematode-associated bacteria at the genus level. (PNG 578 kb)

Additional file 9: **Figure S4.** Expression and hemolytic activity of P450139. (a) Coomassie-stained SDS-PAGE showing heterologous expression and solubility of wild type and 8His-tagged constructs of the P450139 protein. 20 μl of whole cell extract (WCE), supernatants of low spin (LS; 5 min at 5000 g) and high spin (HS; 30 min at 16000 g) bacterial lysate were loaded on a gel. CGL2 was used as positive control for IPTG-induced expression and solubility. (b) The P450139-8His construct was produced in *E.coli Bl21* and 12 μg of Ni-NTA purified protein loaded onto the SDS-PAGE along with 20 μl of WCE. (c) Potential hemolytic activity of purified P450139 proteins was assayed with horse erythrocytes. Triton X-100 and BSA were used as positive and negative controls, respectively. Dunnett’s multiple comparisons test was used for statistical analysis. Error bars represent standard deviation of three biological replicates. (PNG 1049 kb)

## Abbreviations

BP: Biological process
CCL2: *Coprinopsis cinerea* lectin 2
CCTX2: *Coprinopsis cinerea* toxin 2
CGL2: *Coprinopsis cinerea* galectin 2
DEGs: Differentially expressed genes
DUF: Domain of unknown function
EV: Empty vector
FC: Fold change
FDR: False discovery rate
FGCZ: Functional Genomics Center Zurich
FNI: Fungus-nematode interaction
GO: Gene ontology
IPTG: Isopropyl β-D-1-thiogalactopyranoside
JGI: Joint Genome Institute
MEA: Malt extract agar
MF: Molecular function
NGM: Nematode growth medium
OTUs: Operational taxonomic units
PBS: Phosphate-buffered saline
ROS: Reactive oxygen species
VCC: *Vibrio cholera* cytolysin
