# Supplementary material for: Combining microfluidics and RNA-sequencing to assess the inducible defensome of a mushroom against nematodes
Source: BMC Genomics. 2019 Mar 25;20:243. doi: 10.1186/s12864-019-5607-3 (PMC6434838; doi:10.1186/s12864-019-5607-3)
Supplement: Supplementary file 1 — Table S1. Used organisms. Used organism in this study including strain and source information. (DOCX 13 kb) [file 12864_2019_5607_MOESM1_ESM.docx]

**Table S1: Used organisms**

| **Name** | **Strain** | **Source/Reference** |
| --- | --- | --- |
| *Caenorhabditis elegans* | N2 | Caenorhabditis Genetics Center (CGC) |
| *Caenorhabditis briggsae* | AF16 | Caenorhabditis Genetics Center (CGC) |
| *Caenorhabditis tropicalis* | JU1373 | Caenorhabditis Genetics Center (CGC) |
| *Halicephalobus gingivalis* | Environmental isolate | Pamela Fonderie, Ghent University, Belgium |
| *Pristionchus pacificus* | PS312 | Iain Wilson, BOKU, Vienna, Austria |
| *Aphelenchus avenae* | Standard lab strain | Richard Sikora, University of Bonn, Germany |
| *Aedes aegypti* | Rockefeller | Pie Müller, Swiss Tropical and Public Health Institute, Basel, Switzerland |
| *Coprinopsis cinerea* | AmutBmut | (Swamy et al., 1984) |
| *Botrytis cinerea* | BC-3 | Paul W. Sternberg, California Institute of Technology, Pasadena, USA |
| *Escherichia coli* | DH5α |  |
| *Escherichia coli* | BL21(DE3) | Novagen |
| *Escherichia coli* | OP50 | Hengartner laboratory (University of Zürich) |
