# Supplementary figures and images for: Combining microfluidics and RNA-sequencing to assess the inducible defensome of a mushroom against nematodes
Source: BMC Genomics. 2019 Mar 25;20:243. doi: 10.1186/s12864-019-5607-3 (PMC6434838; doi:10.1186/s12864-019-5607-3)

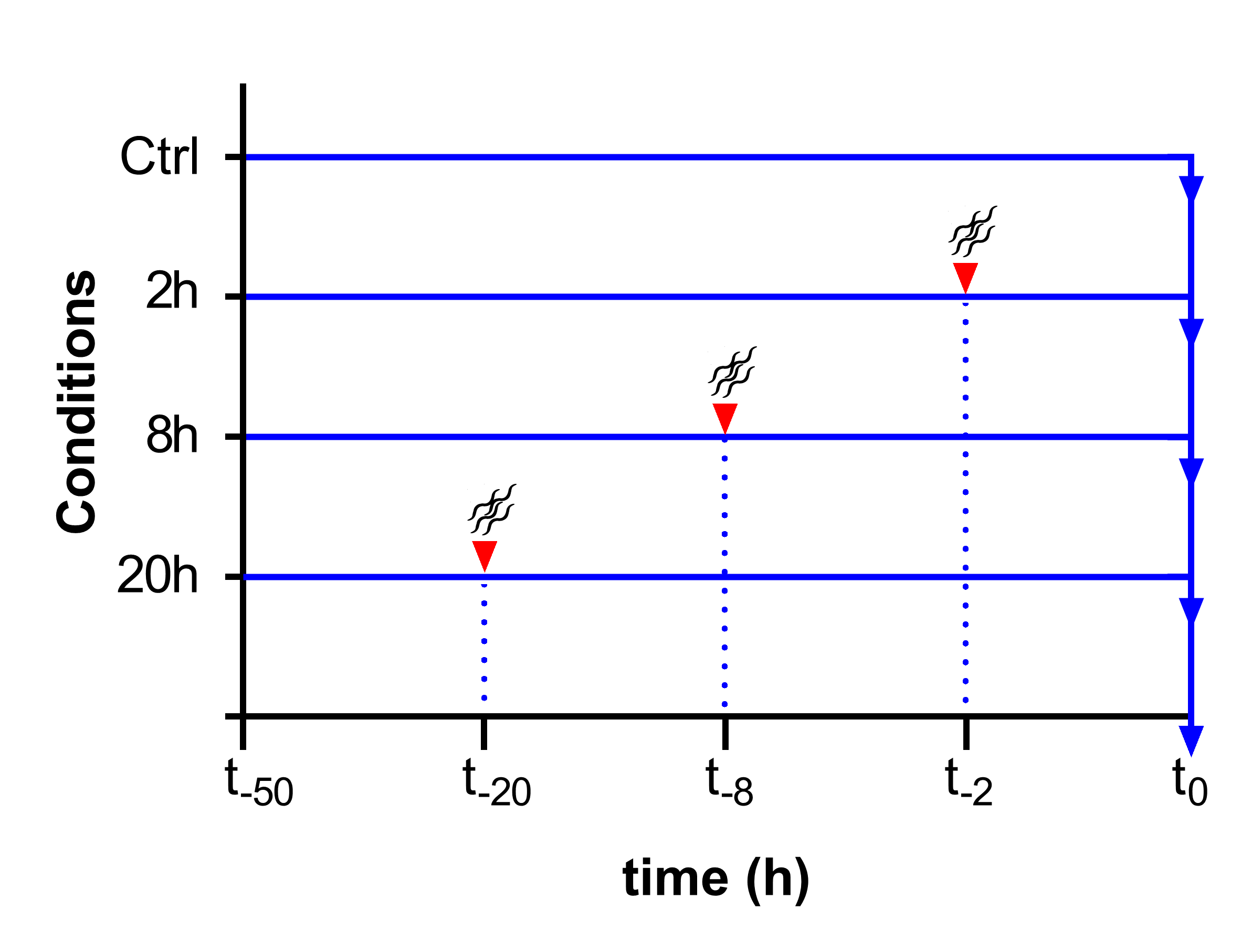

Supplement: Supplementary file 2 — Figure S1. Nematode-fungus challenge experiment setup and RNA extraction. Microfluidic devices were inoculated with a small C. cinerea plug and incubated for 30 h. Around ten worms were added to the confrontation area of the microfluidic device at three different time points i.e. 2 h, 8 h and 20 h. The time point when nematodes were added are indicated with a red triangle. The control samples were incubated in the absence of the predator nematodes throughout. All samples were harvested at the same time point (t0) and used for total RNA extraction. Each treatment was performed in three biological replicates. (PNG 70 kb) [file 12864_2019_5607_MOESM2_ESM.png]

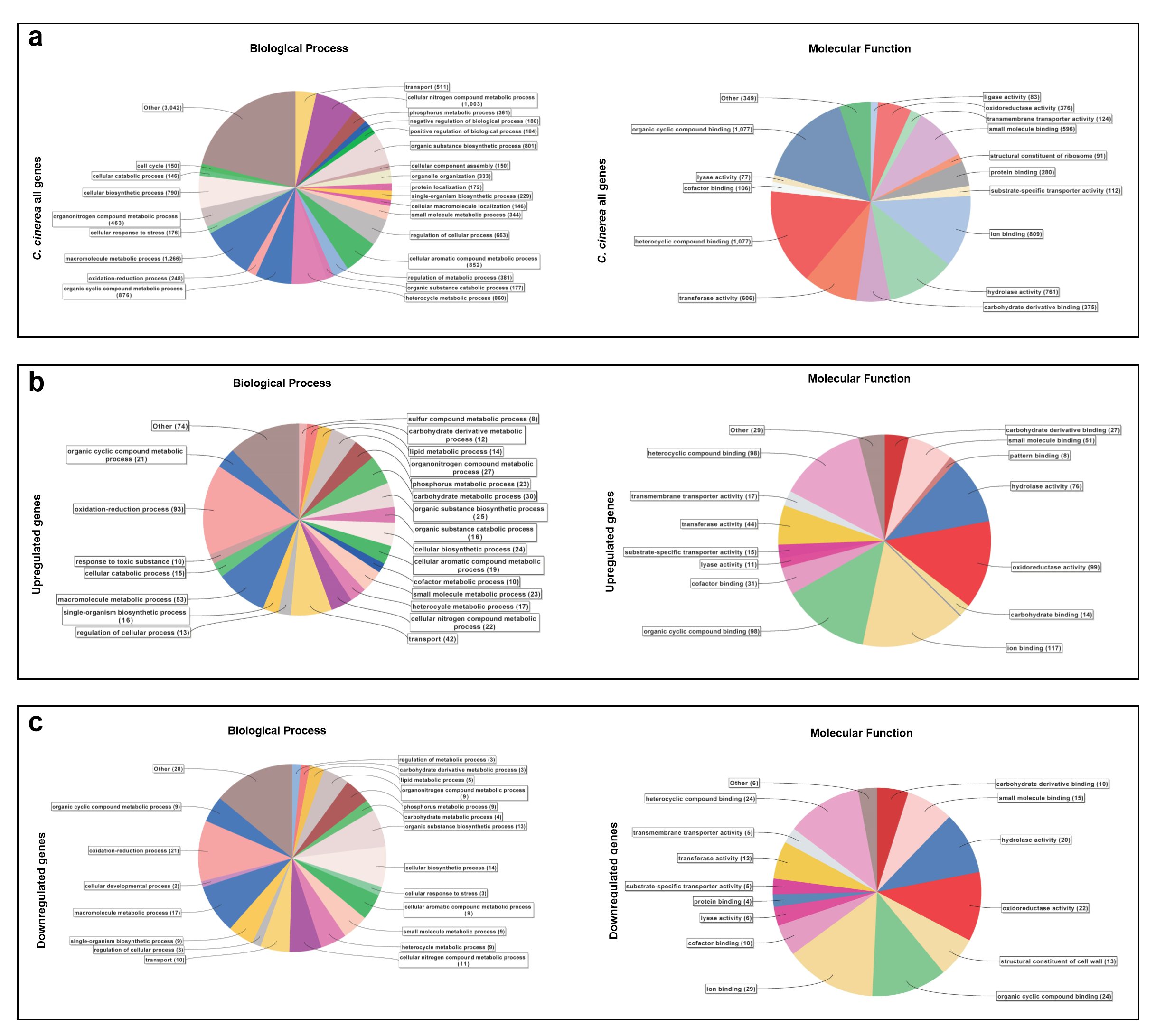

Supplement: Supplementary file 6 — Figure S2. Annotation of DEGs with Blast2GO into functional categories. All (a), upregulated (b) and downregulated (c) genes of C. cinerea due to nematode challenge were annotated with Blast2GO. Categories are grouped by biological process (BP) and molecular function (MF). Numbers indicate the number of genes assigned to each GO term. (PNG 1140 kb) [file 12864_2019_5607_MOESM6_ESM.png]

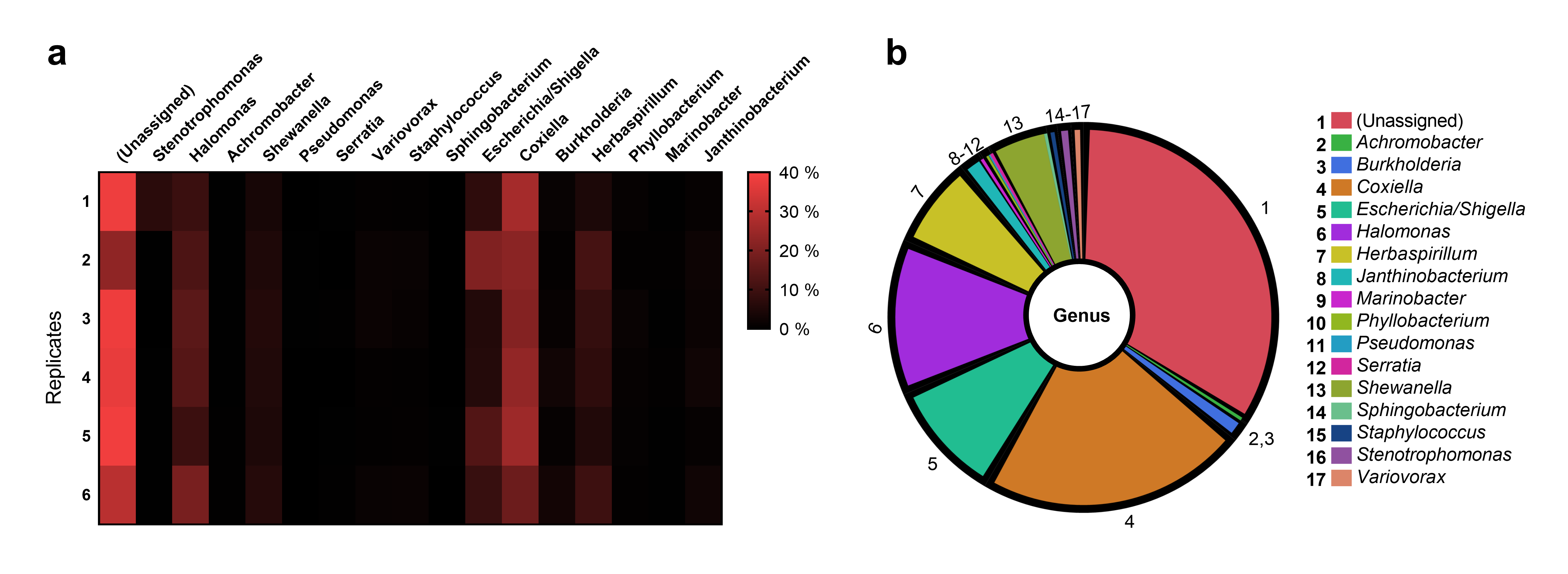

Supplement: Supplementary file 8 — Figure S3. Analysis of 16 rRNA-based associated bacteria of A. avenae. Description: (a) Heat map showing 16S rDNA based profile of A. avenae associated bacteria for each of the six biological replicates. The figure legend represents the percentage of assigned OTUs to each genus of bacteria. (b) The pie chart represents the composition of nematode-associated bacteria at the genus level. (PNG 578 kb) [file 12864_2019_5607_MOESM8_ESM.png]

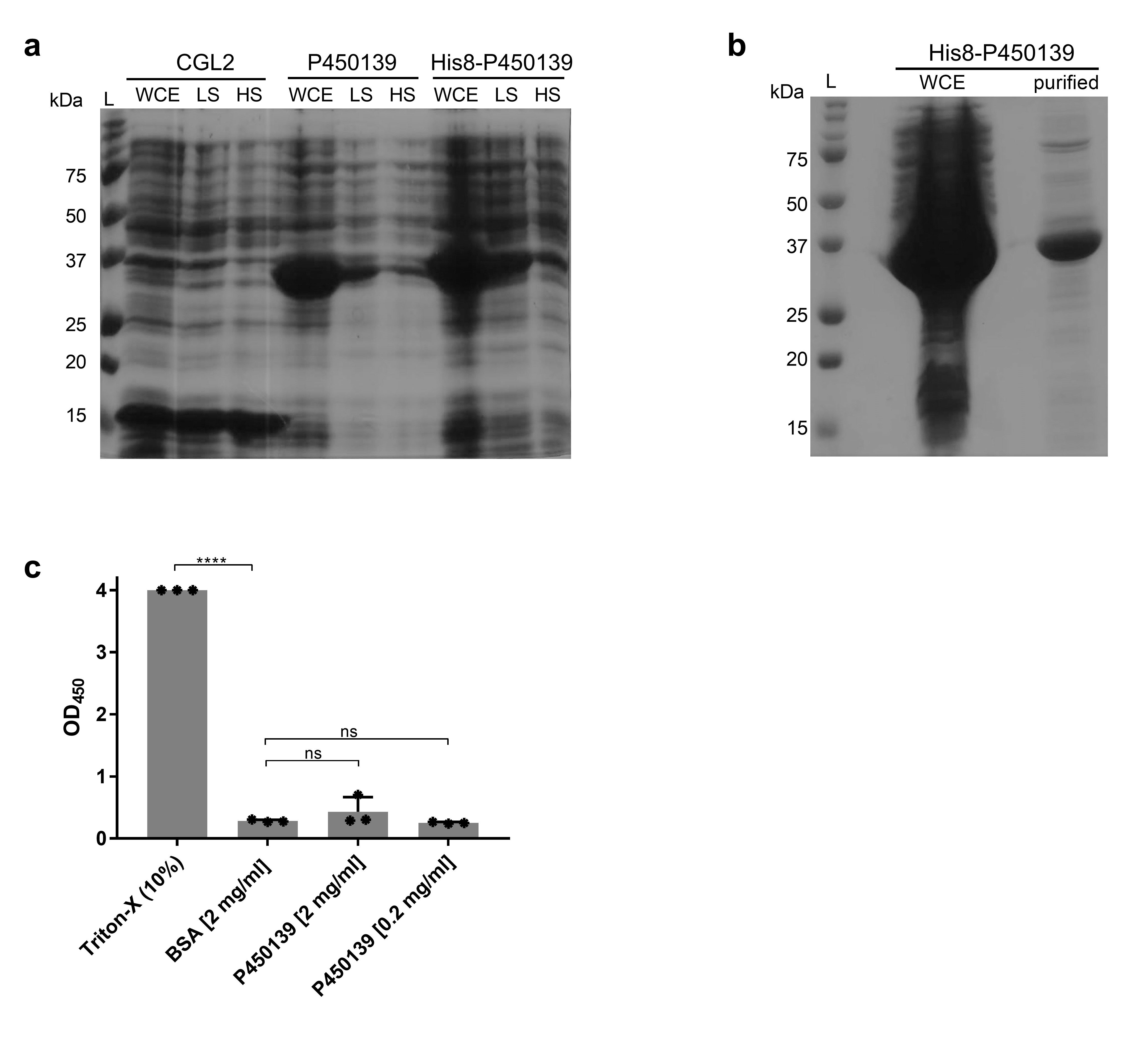

Supplement: Supplementary file 9 — Figure S4. Expression and hemolytic activity of P450139. (a) Coomassie-stained SDS-PAGE showing heterologous expression and solubility of wild type and 8His-tagged constructs of the P450139 protein. 20 μl of whole cell extract (WCE), supernatants of low spin (LS; 5 min at 5000 g) and high spin (HS; 30 min at 16000 g) bacterial lysate were loaded on a gel. CGL2 was used as positive control for IPTG-induced expression and solubility. (b) The P450139-8His construct was produced in E.coli Bl21 and 12 μg of Ni-NTA purified protein loaded onto the SDS-PAGE along with 20 μl of WCE. (c) Potential hemolytic activity of purified P450139 proteins was assayed with horse erythrocytes. Triton X-100 and BSA were used as positive and negative controls, respectively. Dunnett’s multiple comparisons test was used for statistical analysis. Error bars represent standard deviation of three biological replicates. (PNG 1049 kb) [file 12864_2019_5607_MOESM9_ESM.png]
