# Supplementary material for: Combining microfluidics and RNA-sequencing to assess the inducible defensome of a mushroom against nematodes
Source: BMC Genomics. 2019 Mar 25;20:243. doi: 10.1186/s12864-019-5607-3 (PMC6434838; doi:10.1186/s12864-019-5607-3)
Supplement: Supplementary file 3 — Table S2. Primer list. List of the primers used in this study. (DOCX 12 kb) [file 12864_2019_5607_MOESM3_ESM.docx]

**Table S2: Primer list**

| **Name** | **Sequence (5’-3’)** |
| --- | --- |
| P450139forNdeI | GGCGCATATGCCAGAAGACACCAAGAACCTCTACGACAGCATC |
| P450139revNotI | AATGCGGCCGCTCATTCCGTCTTGGGGCGAG |
| P450139forHis8 | CACCACCACCACGAAGACACCAAGAACCTCTACG |
| P450139revHis8 | ATGATGATGATGTGGCATATGTATATCTCCTTCT |
| 515f | TCGTCGGCAGCGTCAGATGTGTATAAGAGACAGNNNNNGTGCCAGCMGCCGCGGTAA |
| 806r | GTCTCGTGGGCTCGGAGATGTGTATAAGAGACAGNNNNNGGACTACHVGGGTWTCTAAT |
