# Supplementary material for: Combining microfluidics and RNA-sequencing to assess the inducible defensome of a mushroom against nematodes
Source: BMC Genomics. 2019 Mar 25;20:243. doi: 10.1186/s12864-019-5607-3 (PMC6434838; doi:10.1186/s12864-019-5607-3)
Supplement: Supplementary file 4 — Table S3. RNA sequencing read counts and alignment statistics. As stated in the title. (DOCX 15 kb) [file 12864_2019_5607_MOESM4_ESM.docx]

**Table S3:** RNA sequencing read counts and alignment statistics

| Samples | Total raw reads | Mapped reads | Mapped reads (%) | Genomic features (%)* |
| --- | --- | --- | --- | --- |
| Ctrl_1 | 33,736,525 | 28,931,231 | 85.8 | 80.7 |
| Ctrl_2 | 42,267,578 | 36,248,399 | 85.8 | 80.8 |
| Ctrl_3 | 45,756,430 | 39,568,173 | 86.5 | 80.5 |
| 2h_1 | 31,160,742 | 27,255,939 | 87.5 | 79.7 |
| 2h_2 | 47,247,117 | 39,506,827 | 83.6 | 81.1 |
| 2h_3 | 36,702,592 | 31,494,884 | 85.8 | 80.3 |
| 8h_1 | 34,036,193 | 29,951,723 | 88.0 | 81.7 |
| 8h_2 | 30,921,637 | 26,427,852 | 85.5 | 81.2 |
| 8h_3 | 39,23,1376 | 33,172,543 | 84.6 | 81.5 |
| 20h_1 | 36,737,179 | 31,889,797 | 86.8 | 80.3 |
| 20h_2 | 53,957,132 | 45,826,904 | 84.9 | 81.4 |
| 20h_3 | 37,693,002 | 31,437,156 | 83.4 | 79.8 |

*Percentage of genes with reads above threshold 10
